# Personalized Antifibrotic Therapy in CKD Progression

**DOI:** 10.3390/jpm14121141

**Published:** 2024-12-05

**Authors:** Charlotte Delrue, Michele F. Eisenga, Joris R. Delanghe, Marijn M. Speeckaert

**Affiliations:** 1 Department of Nephrology, Ghent University Hospital, 9000 Ghent, Belgium; charlotte.delrue@ugent.be; 2 Division of Nephrology, Department of Internal Medicine, University Medical Center Groningen, University of Groningen, 9712 CP Groningen, The Netherlands; m.f.eisenga@umcg.nl; 3 Department of Diagnostic Sciences, Ghent University, 9000 Ghent, Belgium; joris.delanghe@ugent.be; 4 Research Foundation-Flanders (FWO), 1000 Brussels, Belgium

**Keywords:** chronic kidney disease, kidney fibrosis, antifibrotic therapy, biomarkers, precisions medicine

## Abstract

Chronic kidney disease (CKD) is a chronic disorder characterized by kidney fibrosis and extracellular matrix accumulation that can lead to end-stage kidney disease. Epithelial-to-mesenchymal transition, inflammatory cytokines, the TGF-β pathway, Wnt/β-catenin signaling, the Notch pathway, and the NF-κB pathway all play crucial roles in the progression of fibrosis. Current medications, such as renin–angiotensin–aldosterone system inhibitors, try to delay disease development but do not stop or reverse fibrosis. This review emphasizes the growing need for tailored antifibrotic medications for CKD treatment. Precision medicine, which combines proteomic, metabolomic, and genetic data, provides a practical way to personalize treatment regimens. Proteomic signatures, such as CKD273, and genetic markers, such as *APOL1* and *COL4A5*, help in patient stratification and focused therapy development. Two recently developed antifibrotic medications, nintedanib and pirfenidone, have been proven to diminish fibrosis in preclinical animals. Additionally, research is being conducted on the efficacy of investigational drugs targeting CTGF and galectin-3 in the treatment of kidney fibrosis.

## 1. Introduction

Chronic kidney disease (CKD) affects millions of individuals worldwide and is a progressive disease that can lead to end-stage kidney disease (ESKD). In high- and middle-income countries, diabetes mellitus, arterial hypertension, and primary glomerulopathies are predominant causes of CKD. However, infectious diseases and other local risk factors significantly contribute to the prevalence of CKD in low-income areas [1]. The prevalence of CKD is expected to increase further internationally, posing significant public health and economic challenges as care costs escalate, including dialysis and kidney transplants [2]. Patients with early-stage disease are often asymptomatic. One of the comorbidities associated with CKD that increases the risk of disease progression is cardiovascular disease, which is the primary cause of death [1].

Modern CKD treatment attempts to reduce proteinuria and treat associated conditions such as arterial hypertension and diabetes mellitus. The renin–angiotensin–aldosterone system (RAAS) inhibitors and mineralocorticoid receptor antagonists (MRAs) are not entirely successful in stopping the progression of kidney function decline and kidney fibrosis [3]. In recent years, sodium-glucose cotransporter 2 (SGLT2) inhibitors, such as dapagliflozin and empagliflozin, have become standard components of CKD treatment. SGLT2 inhibitors, which were originally developed to help diabetics control their blood sugar levels, have significant kidney protective effects, including the ability to reduce kidney fibrosis. SGLT2 inhibitors reduce inflammation, oxidative stress, and capillary damage, thereby ameliorating kidney fibrosis through a vascular endothelial growth factor (VEGF)-dependent mechanism [4]. Recent research has also indicated the potential benefits of GLP-1 receptor agonists (GLP1-RA) in the treatment of CKD. In the FLOW trial, semaglutide demonstrated renal-protective properties in people with type 2 diabetes (T2D) and CKD [5].

The role of kidney fibrosis in the development of CKD remains debatable [6]. Fibrosis can be caused by inflammation, oxidative stress, or cellular damage, or it can be the direct cause of CKD. In preclinical models, fibrosis can occur before other pathological alterations, such as inflammation and tubular atrophy, suggesting that fibrosis may be the first stage of the disease [7]. Disparities between animal models and human pathology have made it difficult to apply these findings to people [8]. One of the major challenges is the complexity of the fibrotic pathways involved in CKD. Multiple overlapping processes, including inflammation, oxidative stress, cell cycle arrest, and drive fibrosis, make it difficult for single-target therapies to show significant benefits. In clinical trials, attempts to block important factors such as transforming growth factor-beta (TGF-β), a key regulator of fibrosis, have failed because of unfavorable side effects and ineffective targeting of all pathogenic processes [9]. However, fibrosis is still a major target for CKD treatments despite these complications since it is the common end pathway of most kidney injuries, regardless of the initial cause. Fibrosis is closely correlated with the probability of CKD progressing to end-stage kidney disease (ESKD), as demonstrated by preclinical models [10]. If halting or reversing fibrosis directly improves clinical outcomes for patients with CKD, further study is needed to reach that conclusion.

One of the most significant barriers to treating kidney fibrosis is the lack of reliable and non-invasive fibrosis evaluation tools. The current gold standard, kidney biopsies, is invasive with procedure-related risks and impractical for routinely tracking the advancement of fibrosis. Furthermore, only a tiny sample of kidney tissue is obtained from biopsies, which increases the risk of sampling errors and understates the severity of fibrosis [11]. New non-invasive approaches, like biomarker testing and sophisticated imaging techniques, provide hope for enhancing the clinical evaluation of fibrosis. The capacity of methods like diffusion-weighted imaging and magnetic resonance imaging (MRI)-based elastography to identify alterations in kidney tissue stiffness, a property that is correlated with fibrotic burden, is being investigated. These techniques, in addition to the use of urine biomarkers (such as collagen degradation fragments), demonstrate potential in preclinical models but need more human study validation [12].

Tailored therapies and personalized medicine represent an important approach to healthcare, with interventions precisely created to address the unique needs of individual patients. These tactics are becoming more widely acknowledged across medical fields, drawing on insights into molecular circuits, genetic markers, and patient-specific data. Beyond the molecular level, demographic and epidemiological considerations are significant in developing personalized treatment options. Such approaches are especially relevant when dealing with complex diseases such as CKD, which necessitate individualized therapy strategies due to a range of risk factors and disease mechanisms. Personalized medicine has demonstrated the ability to improve therapeutic efficacy while decreasing adverse effects, making it an essential component of current medical innovation. The fact that the effectiveness of current drugs can vary depending on the characteristics of the patient illustrates this. For example, statins, which are recommended to reduce cholesterol levels in CKD patients, are not always effective, particularly for those on dialysis [13]. This indicates that a specific approach may be required to determine which patients may benefit the most from a given treatment. The “one-size-fits-all” approach to treating CKD offers some possible alternatives, such as precision medicine, which tailors medicines to the genetics of patients, biomarkers, and environmental factors. This technique aims to enhance patient outcomes by reducing side effects and increasing efficacy [14]. Precision medicine seeks to identify genetic variations, biomarkers, and epigenetic variables that may impact the therapeutic response or susceptibility to fibrosis in CKD. The predictive power of genetic markers, such as circulating tumor necrosis factor receptors (TNFR), in the development of CKD associated with diabetic nephropathy has been demonstrated [15]. Furthermore, precision medicine utilizes omics technologies such as proteomics, metabolomics, and genomics to better understand the distinct biological pathways driving CKD progression in each patient [16]. This approach may lead to the development of personalized treatment. Novel antifibrotic drugs, including gene- and microRNA-based therapies, are being investigated for their ability to selectively target fibrotic pathways and halt CKD progression in high-risk individuals [17]. Moreover, methods that incorporate patient-specific data, such as biomarkers, kidney function, and medical history, can assist physicians in modifying treatment strategies and increasing the likelihood of preventing the development of ESKD [18].

The purpose of this review is to highlight the importance of personalized antifibrotic therapies in the treatment of CKD by discussing the current limitations of existing treatments, the role of precision medicine, and emerging therapeutic strategies targeting fibrosis-related pathways.

## 2. Mechanisms of Kidney Fibrosis in CKD

### 2.1. Key Molecular Pathways

Kidney fibrosis causes structural defects and kidney function loss due to excessive accumulation of extracellular matrix (ECM) in the kidney. This is the last common pathway found in almost all CKD cases. Several molecular processes, such as transforming growth factor-beta (TGF-β) signaling, inflammatory cytokine responses, and epithelial-to-mesenchymal transition (EMT), are responsible for kidney fibrosis (Table 1).

#### 2.1.1. Epithelial-to-Mesenchymal Transition and Chronic Inflammation

The EMT is a major factor in the development of kidney fibrosis. During EMT, tubular epithelial cells lose epithelial properties, such as cell polarity and adhesion, and gain mesenchymal properties, such as greater migratory potential and the ability to create ECM components such as fibronectin and collagen. These cells eventually enter the interstitial space, where they undergo myofibroblast differentiation and aid in the fibrotic process [21]. In addition to being associated with tubulointerstitial fibrosis, EMT plays a role in glomerulosclerosis. Proteinuria and advancement of glomerular fibrosis are caused by podocytes undergoing EMT-like alterations, loss of foot processes, and separation from the glomerular basement membrane in cases of glomerular damage [23].

TGF-β is the main inducer of EMT by triggering pathways that are both Smad-dependent and independent. Mesenchymal markers such as vimentin and α-smooth muscle actin increase in response to Smad2/3 activation, but epithelial markers such as E-cadherin decrease. EMT also involves the activation of non-Smad pathways such as phosphatidylinositol 3-kinase (PI3K)/Akt and MAPK, which facilitate the fibrotic response [24]. Targeting key EMT mediators or limiting TGF-β signaling can reduce fibrosis in animal models of CKD [19,25]. However, the use of these insights in clinical settings remains difficult due to the complexity of the disease and the likelihood of off-target consequences [20,23].

Chronic inflammation is another hallmark of CKD, and inflammatory cytokines play an important role in the development of kidney fibrosis. Chronic inflammation caused by cytokines, including tumor necrosis factor-alpha (TNF-α), interleukin (IL)-1β, and IL-6, can result in tissue damage and fibrosis. These cytokines perpetuate the fibrotic cycle by activating immune cells, particularly macrophages, and releasing profibrotic factors, such as TGF-β. Macrophages exhibit two distinct functions in CKD, depending on their polarization. Proinflammatory M1 macrophages secrete large quantities of IL-1β and TNF-α. In contrast, M2 macrophages play an important role in tissue repair and possess anti-inflammatory properties. However, in CKD, the balance between these two macrophage states is disturbed, with M1 macrophages prevailing, worsening fibrosis and inflammation. Macrophages also contribute to kidney fibrosis via macrophage–myofibroblast transition (MMT). TGF-β and other cytokines cause macrophages to develop into myofibroblasts, increasing the number of cells that produce ECM [20]. Proinflammatory cytokines can also cause oxidative stress, which can injure the kidneys. Oxidative stress promotes fibrosis by activating fibroblasts, increasing ECM deposition, and converting epithelial cells into myofibroblasts [26].

#### 2.1.2. Hypoxia

Hypoxia, or decreased oxygen availability, is one of the primary causes of kidney fibrosis, especially in CKD. The kidney’s vascular rarefaction and other structural abnormalities restrict oxygen flow, increasing hypoxia and initiating fibrotic pathways. The hypoxic environment in kidney tissue triggers a series of cellular reactions that worsen organ failure and lead to fibrosis.

Hypoxia induces fibrosis by stabilizing hypoxia-inducible factors (HIFs), including HIF-1α and HIF-2α. These variables influence genes involved in erythropoiesis, metabolic adaptability, and angiogenesis. HIF-1α promotes the expression of profibrotic genes like TGF-β and connective tissue growth factor (CTGF), resulting in ECM deposition and fibroblast activation. Furthermore, hypoxia-induced oxidative stress damages renal epithelial cells and causes EMT [27].

#### 2.1.3. TGF-β Signaling

TGF-β, described as the “master regulator” of kidney fibrosis, is critical in the development of CKD. TGF-β1 is the isoform most commonly associated with kidney fibrosis. TGF-β1 signaling causes fibrosis by activating fibroblasts and inhibiting matrix metalloproteinases (MMPs), which break down the ECM [20]. In addition to fibroblasts, podocytes and endothelial cells, which are crucial in preserving glomerular integrity, are also affected by TGF-β. Specifically, TGF-β induces podocyte loss by apoptosis or separation from the glomerular basement membrane [23].

TGF-β can activate non-canonical pathways, exacerbating the fibrotic response along with the Smad pathway. These pathways include Ras homologous (Rho)-like guanosine triphosphatase (GTPase) signaling, phosphoinositide 3-kinase (P3K)/protein kinase B (Akt), and mitogen-activated protein kinase (MAPK) [20]. TGF-β1 regulates the expression of target genes [*ACTA2* (*α-SMA*), *COL1A1* and *COL3A1*, *CTGF* (*CCN2*), *PAI-1* (*SERPINE1*), *TIMP1*, and *TIMP2*], which increase myofibroblast activation and ECM synthesis, and prevent ECM breakdown by binding to its receptors and activating Smad2 and Smad3. These proteins then translocate to the nucleus to regulate gene expression. This leads to the deposition of fibrotic tissue in the tubulointerstitium and glomeruli. TGF-β stimulates EMT, which allows myofibroblasts to proliferate in the interstitial space, causing tubular epithelial cell death and fibroblast activation [19].

Inhibition of TGF-β signaling has been demonstrated to diminish fibrosis in animal models, but its participation in other physiological systems, such as immune control and tissue homeostasis, makes translating this to humans problematic [28]. Recent evidence suggests that noncoding RNAs, such as microRNAs, alter TGF-β signaling by regulating post-transcriptional gene expression and fibrosis severity [20].

#### 2.1.4. Wnt/β-Catenin Pathway

The Wnt/β-catenin pathway is a remarkably conserved pathway that mediates several biological processes, including cell survival, proliferation, and differentiation. In kidney fibrosis, abnormal activation of the Wnt/β-catenin pathway increases fibroblast activity and ECM formation. The Wnt/β-catenin pathway is initiated by the binding of Wnt proteins to frizzled receptors on the cell surface, leading to stabilization and accumulation of β-catenin in the cytoplasm. After stabilization, β-catenin moves to the nucleus and interacts with transcription factors to promote the expression of target genes linked to fibrosis, including α-smooth muscle actin (*α-SMA*), collagen, and fibronectin. Stimulation of this route causes fibroblasts to transform into myofibroblasts, which are essential for the creation of ECM and kidney fibrosis [29]. Wnt/β-catenin overactivity is linked to glomerulosclerosis and tubulointerstitial fibrosis, which are two conditions that impair kidney function. These fibrotic processes have been demonstrated to be attenuated by inhibition of the Wnt/β-catenin pathway, indicating that this route may be a viable therapeutic target [30]. Drugs based on the Wnt/β-catenin system (ICG-001 and its derivatives, Wnt-C59, poricoic acid ZG, and poricoic acid ZH) have been developed to prevent fibrosis in CKD [31,32,33]. Preclinical studies have suggested that altering Wnt ligands or inhibiting β-catenin signaling may reduce fibrotic indicators. Focusing on this route may slow or stop the progression of kidney fibrosis in patients with CKD [34].

#### 2.1.5. Notch Pathway

The Notch signaling system, a critical regulator of cell fate, influences a variety of processes, such as cell division, proliferation, and death. It has a significant impact on the progression of kidney fibrosis, particularly in patients with CKD. Activation of Notch signaling enhances fibrogenic processes by increasing ECM synthesis and myofibroblast activation. The Notch signaling pathway is activated when a Notch ligand, such as a Jagged or Delta-like ligand, binds to a Notch receptor on the cell surface. When this contact causes proteolytic cleavage of the Notch receptor, the Notch intracellular domain (NICD) is released. NICD translocates to the nucleus, where it associates with the transcription factor CSL (CBF1, Suppressor of Hairless, Lag-1), leading to the transcription of genes that promote fibrosis, such as *CTGF* and *α-SMA* [35]. Notch signaling suppression reduces fibrosis and EMT, making it a potential therapeutic target for CKD [36]. Blocking the Notch pathway has shown promise in preclinical CKD models. Experiments indicate that inhibitors of the Notch signaling cascade, such as γ-secretase inhibitors (GSIs), can prevent the production of the Notch intracellular domain (NICD), improve kidney function, and reduce fibrosis. In mice models of kidney fibrosis, dibenzazepine is effective by blocking the TGF-β/Smad2/3 signaling pathway, which lowers collagen and α-smooth muscle actin, two important indicators of fibrosis [37]. Furthermore, the prodrug Ac-γ-Glu-γ-secretase inhibitor 13a has been created to target the kidney specifically by taking advantage of the increased production of particular activating enzymes in pathological conditions. In models of acute kidney injury, this drug decreases Notch1 expression and its downstream fibrotic pathways, providing nephroprotective effects while minimizing systemic impact [38]. Moreover, RO4929097, though primarily investigated in ocular fibrosis, also shows a significant ability to inhibit EMT via Notch and extracellular signal-regulated kinases (ERK)1/2 signaling pathways, thereby reducing fibrotic progression. This dual route inhibition demonstrates the ability of GSIs, like RO4929097, to regulate fibrotic responses in kidney disease [39].

#### 2.1.6. NF-κB Pathway

Kidney fibrosis is also linked to the nuclear factor kappa-light-chain-enhancer of activated B cell (NF-κB) pathway, which regulates inflammation and immunological responses. It controls the expression of several proinflammatory chemokines, adhesion molecules, and cytokines that influence kidney fibrosis and inflammation. Proinflammatory stimuli, including TNF-α and IL-1β, bind to cell surface receptors, phosphorylate IκB proteins, and inhibit NF-κB. Subsequently, these proteins are broken down. This mechanism activates the NF-κB pathway, which results in increased inflammation and kidney fibrosis. After release from IκB, NF-κB enters the nucleus and activates genes involved in inflammation, fibrosis, and cell survival. The activation of fibroblasts and recruitment of immune cells (macrophages) increases the formation of ECM components, which eventually leads to glomerulosclerosis and tubulointerstitial fibrosis [40,41]. Targeting the NF-κB pathway may minimize kidney fibrosis. Inhibitors of NF-κB signaling can reduce fibrosis and inflammation. In animal models of CKD, peptide inhibitors or small compounds that block IKK activation are required for NF-κB activation, lowered kidney fibrosis, and improved kidney function [42].

### 2.2. Genetic and Environmental Risk Factors

#### 2.2.1. Role of Genetics in Fibrosis Susceptibility

Genome-wide association studies (GWAS) and gene-linkage analyses have identified numerous genetic variants associated with an increased risk of CKD. Polymorphisms related to apolipoprotein L1 (*APOL1*), transforming growth factor-beta receptor (*TGFBR*)2, and *MMP1* have been associated with an increased risk of developing kidney fibrosis. These genetic variations may influence important mechanisms such as ECM alterations and inflammation [7]. In addition to GWAS discoveries, animal models have provided new insights into the genetic basis of fibrosis [7,43]. For example, different strains of rats and mice have varying susceptibilities to kidney fibrosis, implying that genetic composition is an important factor in determining fibrotic reactions. While the precise genetic switches that dictate the course of fibrosis remain unknown, emerging evidence suggests that genetic predisposition plays an important role in determining the rate at which CKD progresses in different individuals [44].

#### 2.2.2. Environmental Triggers

Environmental variables play significant roles in the development of fibrosis and CKD. Chronic exposure to pollutants such as pesticides, NSAIDs, and heavy metals has been linked to an increased risk. In addition, heat fatigue and dehydration have been associated with an unexplained cause of CKD, particularly among agricultural workers. These variables may exacerbate kidney injury and accelerate fibrosis by inducing chronic inflammation and oxidative stress [45]. Understanding the onset of CKD requires an investigation of the relationship between hereditary risk and environmental exposure. For instance, individuals with genetic variations that predispose them to fibrosis may experience more severe kidney damage when exposed to environmental triggers such as pollution or pesticides. Consequently, this gene–environment interaction suggests that tailored preventive strategies addressing both genetic and environmental risk factors could delay CKD onset [43].

## 3. Experimental Therapies in Kidney Fibrosis

### 3.1. Pirfenidone and Nintedanib as Promising Candidates

Unfortunately, unlike idiopathic pulmonary fibrosis (IPF), no antifibrotic drug has been approved specifically for CKD. Nonetheless, preclinical and early-stage clinical trials for kidney fibrosis have shown promise for drugs approved for IPF treatment, including pirfenidone and nintedanib (Table 2).

The antifibrotic drug pirfenidone was initially licensed for IPF management. Pirfenidone inhibits the production of cytokines that induce fibrosis, specifically TGF-β and interleukin-1β, which is required for progression across all organs. It reduces oxidative stress by scavenging reactive oxygen species (ROS), which are another important element in fibrosis. In clinical trials with IPF patients, pirfenidone improves survival rates and reduces lung function decline. Its efficacy in treating CKD is currently under investigation. However, kidney fibrosis is closely associated with the molecular pathways it targets [46,51].

Nintedanib is another approved IPF treatment option. It targets multiple cellular processes involved in fibrosis, giving it a particular advantage over other antifibrotic medications [52]. Nintedanib suppresses tyrosine kinases, including those that function as platelet-derived growth factor (PDGF), fibroblast growth factor (FGF), and vascular endothelial growth factor (VEGF) receptors, which play key roles not only in fibrosis but also in inflammation and endothelial cell dysfunction. In addition, it inhibits fibroblast activation, tissue scarring, and ECM production. According to clinical trials, nintedanib helps individuals with IPF experience fewer acute exacerbations and delays the decrease in FVC. Additionally, nintedanib has been studied for its potential antifibrotic effects in CKD [47]. DENNM is a new type of nanocarrier that was created to specifically target fibrotic kidneys. It works by taking advantage of the overexpression of leucine-rich α-2 glycoprotein 1 (LRG1) that occurs during renal fibrosis. An ET peptide that binds to LRG1 decorates the nanocarrier and enables it to concentrate in injured renal cells. When caspase-3 is activated within cells, the nanocarrier liberates the medication nintedanib. Nintedanib slows the evolution of renal fibrosis by inhibiting the TGF-β-Smad2/3 pathway and lowering ECM production. This delivery approach demonstrated effectiveness in both in vitro and in vivo studies, underlining LRG1’s potential as a target for renal fibrosis [53].

These antifibrotic medicines reduce inflammation, hence reducing the detrimental consequences of chronic inflammation, such as oxidative stress and cellular death. Furthermore, they have the ability to affect cellular metabolism by regulating mitochondrial bioenergetics and reducing ROS formation, which can have a broader therapeutic impact than only fibrosis [54].

### 3.2. PRM-151 and Ziritaxestat

PRM-151, commonly known as serum amyloid P or recombinant human pentraxin-2, has been investigated as a potential drug for fibrosis. PRM-151 acts as a naturally occurring protein that modulates immune responses by converting monocytes into a fibrosis-inhibiting phenotype. PRM-151, which inhibits fibroblast activation and ECM deposition, reduced fibrosis in a range of organs, including the kidneys and lungs, in preclinical studies [55]. In phase 2 clinical trials, PRM-151 demonstrated promise in stabilizing lung function and lowering bone marrow fibrosis in patients with IPF and myelofibrosis, respectively. These findings suggested its potential for fibrotic disorders, including fibrosis associated with CKD. The mechanism of PRM-151 may rely on preventing fibrosis by reducing fibrocyte recruitment and inflammatory signaling, which are also critical pathways in kidney fibrosis [56]. However, in the recently published phase III STARSCAPE Trial, no clinical benefit of zinpentraxin alfa over placebo was demonstrated for IPF patients [57]. Moreover, a post hoc analysis indicated that the extreme forced vital capacity (FVC) decline in two placebo patients had skewed the positive results in phase 2.

Ziritaxestat, an inhibitor, targets an enzyme called autotaxin (ATX), which is involved in the formation of lysophosphatidic acid. It has been useful in the treatment of fibrotic diseases. Lysophosphatidic acid signaling promotes fibrosis by stimulating fibroblast activation, inflammation, and ECM deposition. Preclinical investigations have shown that ziritaxestat effectively reduced indicators of inflammation and fibrosis in animal models of systemic sclerosis despite challenges in IPF [58]. Though primarily studied in IPF through phase III trials (ISABELA 1 and ISABELA 2), ziritaxestat’s impact on ATX and LPA pathways suggests that it may have antifibrotic potential in kidney fibrosis as well. However, the ISABELA trials in IPF were terminated early due to a lack of efficacy in improving FVC and secondary endpoints compared to placebo, possibly due to alternative pathways driving fibrosis in these patients [59].

### 3.3. Hypoxia-Inducible Factor-Prolyl Hydroxylase Inhibitors

To reduce fibrosis in CKD, recent research has also focused on therapy options that targeted the hypoxia-inducible factor (HIF) pathway. HIF-prolyl hydroxylase inhibitors (HIF-PHIs) are being studied for their capacity to stabilize HIFs in hypoxic conditions, thereby balancing HIF-mediated pathways and decreasing kidney fibrosis without exacerbating inflammatory or fibrotic responses. HIF-PHIs aim to improve adaptive responses to hypoxia while reducing maladaptive effects that cause fibrosis, providing a unique strategy for delaying CKD progression [22]. HIF-PHIs, such as roxadustat and JTZ-951, are primarily used to treat anemia by increasing erythropoietin (EPO) levels and improving iron metabolism. However, research suggests they may help reduce fibrosis, which is a critical step in the development of CKD [60]. In preclinical models of renal ischemia, roxadustat reduces interstitial fibrosis, perhaps by restricting fibroblast transformation and oxidative damage [61]. The therapeutic concept that HIF-PHIs could decrease CKD progression by stabilizing HIF and modulating fibrotic responses is supported by JTZ-951’s ability to diminish renal fibroblast activation and fibrosis-related variables in vitro [62]. However, some studies suggest that HIF stabilization may not always directly reduce kidney fibrosis, even if it stimulates erythropoiesis. This indicates that HIF-PHIs have limited antifibrotic activity. Pan et al. [63] emphasized the importance of cell-specific considerations in HIF-PHI therapy by demonstrating that HIF activation in pericytes, which are progenitor cells for myofibroblasts, enhanced EPO synthesis but did not significantly reduce fibrosis. These findings emphasize the dual benefits of HIF-PHIs in treating anemia and potentially protecting the kidneys. HIF-PHIs target hypoxia pathways and offer a unique treatment approach for CKD. However, further research is needed to fully understand their antifibrotic effects. With its multi-targeted approach, patients with advanced disease may receive a more comprehensive therapy plan that addresses not only fibrosis but also the concomitant metabolic and inflammatory processes that hasten the course of CKD.

### 3.4. Emerging Targets

#### 3.4.1. Connective Tissue Growth Factor

A variety of newly discovered molecular targets, including connective tissue growth factor (CTGF) and galectin-3, are potential therapeutic targets because of their involvement in the fibrotic pathways. CTGF regulates TGF-β signaling, and because fibrotic kidney disease is characterized by CTGF overexpression, inhibition of this protein may provide a treatment option. Targeting CTGF has been shown in preclinical studies to reduce the deposition of ECM components, such as collagen, and promote kidney scarring. Antisense oligonucleotides targeting CTGF dramatically decreased ECM buildup and interstitial fibrosis in an animal model of unilateral ureteral obstruction (UUO) [64]. The CTGF-targeting monoclonal antibody FG-3019 was evaluated in a phase 1 trial with diabetic kidney disease (DKD). This study found a decrease in albuminuria, suggesting that CTGF inhibition played a role in arresting fibrosis and maintaining kidney function. This appears to be a viable starting point for future research examining its effectiveness in kidney fibrosis [48]. Multiple preclinical models have focused on CTGF [65]. A seminal study showed that antisense oligonucleotides blocking CTGF markedly reduced EMT and fibrosis in mice [66]. According to a phase 1 study, FG-3019 was well tolerated and reduced albuminuria in patients with DKD [48]. These preliminary data suggest that CTGF holds promise as a therapeutic target for kidney fibrosis, although this study was not specifically designed to extensively investigate its efficacy.

#### 3.4.2. Galectin-3

Another key target, galectin-3, is a β-galactoside-binding lectin that promotes fibrosis by enhancing the activity of myofibroblasts. Martínez-Martínez et al. conducted a study on the blockade of galectin-3 in experimental models of kidney damage, showing that inhibition of galectin-3 reduced fibrosis, inflammation, and kidney injury. Modified citrus pectin (MCP) was used to suppress galectin-3 in two normotensive kidney injury models. This intervention enhanced kidney function and lowered the levels of fibrotic markers such as collagen and TGF-β [49]. Furthermore, MCP increased glutathione levels, decreased malondialdehyde levels, and increased catalase activity in type 2 diabetic mice with early-stage diabetic nephropathy. MCP showed a significant decrease in the mediators of apoptosis and inflammation, TNF-α, iNOS, TGF-βRII, and caspase-3 [67]. The inhibition of galectin-3 has also been explored in preclinical models. A galectin-3 inhibitor called MCP lessened kidney fibrosis and inflammation in two normotensive animal models of kidney damage. In these mice, galectin-3 inhibition reduced collagen deposition and ECM formation, indicating a potential therapeutic strategy [49]. Furthermore, animal models of fibrosis have illustrated that galectin-3 inhibitors, such as GR-MD-02, greatly reduced inflammation and ECM deposition in the kidneys and other organs. These findings suggest that galectin-3 inhibitors may offer a novel approach to decrease fibrosis in CKD [68].

#### 3.4.3. Homeodomain-Interacting Protein Kinase 2

Recent breakthroughs in the development of homeodomain-interacting protein kinase 2 (HIPK2) inhibitors have demonstrated their potential as anti-kidney fibrosis agents. These inhibitors specifically target the HIPK2 protein, which is involved in a variety of biological processes, such as cell death, differentiation, and fibrosis. HIPK2 has been linked to fibrotic pathways in kidney disease, specifically through the regulation of TGF-β/Smad3, Wnt/β-catenin, Notch, and NF-κB pathways. HIPK2 inhibitors have shown potential in preclinical studies to slow the progression of kidney fibrosis. They function by inhibiting profibrotic signaling pathways, thereby lowering the accumulation of ECM proteins that lead to fibrosis. These inhibitors also aid in the suppression of EMT. Although the development of these inhibitors is still in its early phases, it appears that they will provide a personalized therapeutic option for kidney fibrosis, particularly in DKD and CKD. Subsequent research should focus on improving the effectiveness and selectivity of these inhibitors as well as assessing their long-term impact and safety in medical settings [69].

#### 3.4.4. Lademirsen (miR-21 Antagonist)

The miR-21 antagonist lademirsen has been suggested as one of the most promising novel treatments for renal fibrosis. miRNA-21 (miR-21) is essential for promoting tubular injury, inflammation, and fibrosis. Preclinical research has demonstrated that lademirsen, an inhibitor of miR-21, dramatically reduced inflammation and fibrosis, especially in animals with Alport syndrome, a genetic kidney disease that causes progressive fibrosis. The combination of lademirsen with angiotensin-converting enzyme inhibitors (ACEi) had a synergistic therapeutic effect in animals, as seen by improved kidney function, lower proteinuria, and longer lifespans [70]. In a recent phase 2 clinical trial, the effects of lademirsen therapy were evaluated in adults with Alport syndrome who were at risk of rapid disease progression. While lademirsen had an acceptable safety profile, it did not demonstrate a meaningful clinical effect in slowing kidney function decline [71].

#### 3.4.5. Interleukin-11 (IL-11) Targeting

Interleukin-11 (IL-11) has emerged as another potential target for antifibrotic therapies. IL-11 is a cytokine that plays a key role in promoting fibroblast activation and ECM deposition, contributing to tissue fibrosis. Recent studies have demonstrated that blocking IL-11 signaling pathways in animal models can significantly reduce kidney fibrosis, particularly in CKD caused by diabetic nephropathy. Early research suggests that therapies targeting IL-11 could provide broad-spectrum antifibrotic effects across multiple organs, including the kidneys, lungs, and liver [72].

#### 3.4.6. Pentoxifylline

Pentoxifylline, previously used as a phosphodiesterase inhibitor, has shown promise as a treatment for CKD due to its ability to increase levels of Klotho, a protein with important renoprotective and antifibrotic properties. Klotho changes processes involved in the development of CKD that are linked to oxidative stress, inflammation, and fibrosis. Pentoxifylline enhances soluble Klotho and improves Klotho expression in renal tubular cells, particularly in diabetic kidney disease. Klotho’s anti-inflammatory and antifibrotic effects are demonstrated by its link with improved albuminuria and decreased inflammatory markers such as TNF-α [50].

In experimental CKD models, pentoxifylline has been demonstrated to reduce inflammation and interstitial fibrosis. Pentoxifylline reduced renal fibrosis in rat models of crescentic glomerulonephritis by inhibiting TGF-β1 signaling and Smad2/3 activation, which are crucial for ECM accumulation and fibrotic progression [73]. The significance of pentoxifylline in renal architecture and function is further underscored by its ability to suppress myofibroblast differentiation and collagen synthesis.

Additionally, when used in combination with renin–angiotensin system blockers, pentoxifylline has additive renoprotective advantages, effectively lowering proteinuria and maintaining GFR. According to Kuo et al. [74], this implies that it enhances the current therapies for CKD. Pentoxifylline is a possible supplemental treatment due to its multimodal effects on fibrosis, inflammation, and Klotho expression.

## 4. Personalization of Antifibrotic Therapies

Biomarkers for patient classification based on fibrosis risk and therapy response prediction must be identified and validated before antifibrotic medications can be tailored to patients with CKD. It is difficult to reliably identify patients with early-stage CKD or predict when they will develop fibrosis based on traditional indicators, such as serum creatinine and albuminuria. However, advancements in proteomic, metabolomic, and genomic technologies have made it possible to use new strategies to determine which individuals would benefit the most from certain antifibrotic agents.

### 4.1. Genetic Markers

Genetic biomarkers can indicate the susceptibility of patients to fibrosis and CKD progression. The *APOL1* gene is associated with an increased risk of CKD and its progression to ESKD in people of African origin. Genetic testing for *APOL1* mutations can assist in identifying high-risk individuals who may benefit from aggressive or early treatment [75]. A mutation in the collagen type IV alpha-5 chain (*COL4A5*) gene is a genetic marker of CKD and is associated with Alport syndrome, a hereditary disease marked by kidney dysfunction and kidney fibrosis [76].

Mutations in *COL4A5* cause structural abnormalities in the glomerular basement membrane, which lead to proteinuria, fibrosis, and, ultimately, renal failure. The early diagnosis and focused therapy approaches, such as gene-based medicines and miRNA pathway inhibitors (e.g., lademirsen) that reduce the course of fibrosis, are made possible by the identification of patients with *COL4A5* mutations [70]. miRNAs have been found to be potential biomarkers for CKD and kidney fibrosis. MiRNAs, such as miR-21 and miR-29, have a significant impact on fibrotic processes, including TGF-β signaling and ECM formation. Preclinical models have demonstrated that targeting specific miRNAs can be advantageous, and clinical trials are currently being conducted to determine their therapeutic potential [17].

Patients with mutations in the TGF-β signaling system may respond better to TGF-β receptor inhibitors and other drugs that target this pathway. Pharmacogenomic testing also identifies genetic polymorphisms, such as CYP450 enzyme alterations, that affect drug metabolism [77]. Finally, genetic variants in the *TGFBR2* gene have also been found by GWAS and are linked to CKD and kidney fibrosis [78].

### 4.2. Proteomic and Metabolomic Markers

Proteomic and metabolomic technologies are important in CKD because they allow for a full understanding of the molecular pathways that induce kidney fibrosis. Numerous studies have demonstrated that the key proteomic biomarker, CKD273, which classifies 273 urine peptides, is a reliable predictor of CKD progression. The CKD273 classifier can predict not only CKD progression but also the onset of albuminuria in patients with type 2 diabetes [79]. Studies in CKD patients have demonstrated that CKD273 can identify those at risk of cardiovascular events and kidney graft loss, underscoring its utility in both early-stage CKD and post-transplant care [80]. The ability of this panel to stratify patients and predict their treatment response can help guide personalized therapeutic decisions [81].

The advancement of CKD has also been linked to metabolomic indicators, including alterations in the concentrations of metabolites, such as citrulline, kynurenine, and trimethylamine N-oxide (TMAO). These metabolites are associated with the development of fibrosis and abnormalities in metabolic processes. Treatments that target these metabolic pathways have the potential to reverse CKD progression and reduce fibrosis, as high TMAO levels have been linked to poor renal outcomes [82]. Two intriguing metabolic indicators, sphingomyelin C18:1 and phosphatidylcholine diacyl C38:0, were discovered in a recent machine-learning study. These metabolic markers demonstrated a high predictability for CKD in patients with type 2 diabetes. This study significantly improved CKD risk prediction compared to traditional clinical approaches, attaining an area under the receiver operating characteristic curve of 0.857 by combining these metabolites with clinical data [83].

## 5. Conclusions and Future Perspectives

Antifibrotic therapies that target critical signaling pathways, such as TGF-β, CTGF, and galectin-3, have been shown to reduce fibrosis and slow the course of CKD in preclinical models. Although medications such as pirfenidone and nintedanib are being repurposed for CKD, additional clinical trials are needed to prove their efficacy in this setting. Early-phase trials have shown that antifibrotic medications can lower ECM deposition and inflammation while improving kidney function, providing hope for reversing or stopping fibrosis in patients with CKD. Treating CKD more comprehensively may be possible by combining RAAS inhibitors, MRAs, SGLT-2 inhibitors, GLP1-RA, and antifibrotic medications, which address fibrosis, inflammation, and metabolic dysregulation all at once. The future of tailored antifibrotic therapy will depend on the incorporation of innovative biomarkers and advanced machine-learning algorithms in clinical practice. Multi-omics techniques that combine genomic, proteomic, and metabolomic data are expected to improve patient classification and treatment interventions. Furthermore, AI plays an important role in analyzing complicated datasets and determining the best treatment plans for patients with kidney fibrosis.

## Figures and Tables

**Table 1:** Key Molecular Pathways Involved in Kidney Fibrosis in CKD.

Pathway	Mechanism	Impact on CKD Progression	Ref.
TGF-β signaling	Activates Smad2/3, promotes ECM deposition, inhibits ECM degradation.	Central to driving fibrosis, it contributes to glomerulosclerosis and tubulointerstitial fibrosis.	[19]
Inflammatory cytokines	IL-1β, TNF-α, and IL-6 promote immune cell recruitment and macrophage activation.	Fuels chronic inflammation, stimulates macrophage–myofibroblast transition, worsens fibrosis.	[20]
Oxidative stress	Promotes fibroblast activation, increases ECM deposition.	Enhances fibrosis and exacerbates kidney injury through sustained inflammation and oxidative damage.	[20]
EMT	Tubular epithelial cells lose epithelial traits and gain mesenchymal properties.	Increases ECM production, drives myofibroblast proliferation, contributes to glomerular and tubulointerstitial fibrosis.	[21]
Hypoxia	Activates HIF-1α and induces profibrotic gene expression (e.g., CTGF, TGF-β) under low-oxygen conditions.	Drives fibrotic pathways through HIF stabilization, leading to ECM accumulation and exacerbated renal fibrosis.	[22]

Abbreviations: CTGF, connective tissue growth factor; EMT, epithelial-to-mesenchymal transition; ECM, extracellular matrix; HIF, hypoxia-inducible factor; IL, interleukin; TGF-β, transforming growth factor-beta; TNF-α, tumor necrosis factor-alpha.

**Table 2:** Potential Antifibrotic Therapies for Chronic Kidney Disease.

Therapy	Mechanism of Action	Clinical Status	Efficacy in CKD	Ref.
Pirfenidone	Inhibits fibroblast proliferation, reduces TGF-β activity	Approved for IPF, under investigation for CKD (phase 2)	Shown to reduce fibrosis in preclinical models; phase 2 trials ongoing	[46]
Nintedanib	Inhibits VEGF, FGF, and PDGF receptors, reduces fibroblast activity	Approved for IPF, under investigation for CKD (phase 2)	Preclinical models show promise in reducing ECM accumulation and fibrosis	[47]
CTGF Inhibition (FG-3019)	Blocks connective tissue growth factor (CTGF), reduces ECM deposition	Phase 1 trial in diabetic nephropathy	Early clinical studies show reduced albuminuria and kidney damage	[48]
Galectin-3 Inhibitors (MCP)	Inhibits Galectin-3, reduces myofibroblast activity and ECM production	Preclinical studies	Shown to reduce kidney fibrosis and inflammation in animal models	[49]
Pentoxifylline	Increases Klotho levels, reduces inflammation and fibrosis via TGF-β inhibition	Used off-label in CKD	Demonstrated reduction in fibrosis and inflammation in diabetic and other CKD models	[50]

Abbreviations: TGF-β, transforming growth factor-beta; VEGF, vascular endothelial growth factor; FGF, fibroblast growth factor; PDGF, platelet-derived growth factor; CTGF, connective tissue growth factor; ECM, extracellular matrix; IPF, idiopathic pulmonary fibrosis; CKD, chronic kidney disease; MCP, modified citrus pectin; FG-3019, pamrevlumab.

## Data Availability

Not applicable.
